# Leishmaniosis in Greece: The Veterinary Perspective

**DOI:** 10.3390/pathogens12060769

**Published:** 2023-05-26

**Authors:** Isaia Symeonidou, Georgios Sioutas, Athanasios I. Gelasakis, Constantina N. Tsokana, Elias Papadopoulos

**Affiliations:** 1 Laboratory of Parasitology and Parasitic Diseases, School of Veterinary Medicine, Faculty of Health Sciences, Aristotle University of Thessaloniki, 54124 Thessaloniki, Greece; gsioutas@vet.auth.gr (G.S.); nt.tsokana@live.com (C.N.T.); eliaspap@vet.auth.gr (E.P.); 2 Laboratory of Anatomy and Physiology of Farm Animals, Department of Animal Science, School of Animal Biosciences, Agricultural University of Athens, 11855 Athens, Greece; gelasakis@aua.gr

**Keywords:** Greece, *Leishmania infantum*, dogs, cats, prevalence

## Abstract

Leishmaniosis caused by the protozoon *Leishmania infantum* that is transmitted through the bites of infected phlebotomine sandflies is of major veterinary concern in Greece. The country is endemic with particularly favourable environmental conditions for the spread of this infection. Moreover, Greece remains a popular touristic destination, and the continuous travel of pets raises concern regarding the possible dissemination of infection from endemic to non-endemic areas. Dogs are the main reservoir host, although other animal species, including humans, may also be infected. Canine leishmaniosis manifests as a visceral disease that can result in death if left untreated. Serological and molecular epizootiological studies have confirmed circulation of the parasite in Greek canine and feline populations as well as in other mammals. As a result, constant surveillance and identification of high-risk localities are necessary to establish chemoprophylactic protocols for travelling animals to safeguard animal and public health.

## 1. Introduction

Leishmaniosis, caused by protozoan flagellate of the genus *Leishmania*, is of major veterinary concern in Mediterranean countries [1,2]. The primary causative agent is *Leishmania infantum* (*L. infantum*, syn. *L. chagasi*), which belongs to the *Leishmania donovani* complex [3] and infects various animals in these countries [4]. Dogs are considered the parasite’s primary reservoir host, and it may also infect humans [5,6]. However, the parasite’s presence has also been reported in other animals, such as cats, equids, and lagomorphs [7,8,9]. This protozoon is transmitted to the vertebrate host by the female phlebotomine sandflies of the genus *Phlebotomus*, which are considered the natural vectors of *Leishmania* spp., during blood feeding [10]. Sandflies inject the metacyclic promastigotes into the dog’s skin [11], which interact with the immune system, transform into amastigotes, and are transported via infected macrophages to the regional lymph nodes [12].

The outcome of the infection varies depending on factors such as genetic background and immunocompetency of the host, parasite virulence, and others [12,13,14]. In resistant dogs, the parasites are confined in the skin and lymph nodes, and the infection is self-limited or spread throughout the body. In both cases, the dogs remain asymptomatic since *L. infantum* amastigotes are killed intracellularly. In susceptible animals, the parasites disseminate and trigger immune-mediated mechanisms, leading to the occurrence of clinical disease [5,12]. Canine leishmaniosis (CanL) manifests as a visceral disease, representing the most aggressive form of the disease, and can be life-threatening if not treated [1,2]. Intermediate phenotypes can also be found, e.g., resistant dogs that become susceptible later in their lives or dogs with symptoms that become clinically normal without treatment [5,15]. The balance between susceptibility and resistance is predominantly determined by the host’s adequate cell-mediated immunity [16,17].

## 2. The Case of Greece

Greece is endemic and has favourable environmental conditions, i.e., the warm period of the year is extended from April to November. Since adult females are active during the warm months of the year, every Greek dog that lives outdoors may receive roughly one infectious bite every hour during the night, which will repeatedly occur for several months [18]. This results in such intense infection levels that the prevalence of asymptomatic infection in young dogs that have sustained only one period of sandflies is similar to that of older animals [19]. It has been speculated that this cumulative exposure contributes to the establishment of seropositivity and clinical disease [20].

In addition, Greece is a very popular touristic destination, and travels are particularly intense. Millions of people accompanied by their dogs and cats travel annually to tourist spots throughout the country. A significant association between pet movements and dissemination of *L. infantum* from endemic to non-endemic areas has been previously documented [21]. Over 700 imported cases of CanL have been reported in the last years in traditionally free of the parasite countries of Europe [21]. The above have been exacerbated by the fact that leishmaniosis is regarded as a possible emerging threat for travellers [22], and cases of human leishmaniosis have been sporadically evidenced in tourists that visited countries in the Mediterranean, such as Greece and neighbouring Italy [23,24]. *Leishmania infantum* circulates in Greek touristic regions; thus, dogs travelling with their owners are exposed to the parasite and may pose a great risk of developing future autochthonous cases in their country of residence. Therefore, constant surveillance and identification of high-risk localities are of pivotal importance and assist in establishing chemoprophylactic protocols for travelling animals that will restrict the spread of leishmaniosis.

## 3. Prevalence of *L. infantum* in the Canine Population in Greece

In the Mediterranean basin, CanL is endemic and has a broad distribution. In the Mediterranean counties, seroprevalence rates range depending on the area from 4.7% to 57.1% [25,26], with the latter being dog seropositivity in the Balearic Islands, which up today is the highest seroprevalence reported in Europe [26]. Due to various diagnostic tools, antibody titre cut-off values, inclusion criteria, size of samples as well as the time of sampling, significant variations in seroprevalence estimates are expected, and comparison of any results from these studies must be performed with caution [27,28,29]. Likewise, Greece remains a highly endemic country for CanL, as it has been established by several regional or cross-sectional seroprevalence studies over the years conducted in different parts of the country [19,28,30,31,32,33,34]. In detail, surveys that examined apparently healthy infected animals documented different prevalence estimates of CanL from 12.3% [19] to 24.4% [30]. Most asymptomatic dogs remain seronegative [19,35,36,37,38,39], such that the true prevalence of the infection by *L. infantum* in the Greek canine population is much higher than reported in these surveys. As predicted, when both symptomatic and asymptomatic dogs were included, the prevalence rate reached up to 50.2% [31]. Noteworthy, *L. infantum* was identified as the most prevalent vector-borne agent (15.3%) in canine populations residing in Greek islands, which represent popular touristic spots [34]. The initial results concord with a study that used a specially developed questionnaire along with spatial analysis based on available epizootiological data and predicted that most areas of the country are expected to have seropositivity between 3% and 55% in dogs [32]. In the same frame, a recent countrywide serological screening of a large and representative sample detected specific antibodies against *L. infantum* in 13.8% of the asymptomatic canine population. Samples originated from each prefecture of the country, and notably, in all geographical departments, seropositive animals were detected [33]. The prevalence estimates per prefecture varied significantly (from 0% to 53%), as did the distribution of seropositive dogs across the east–west and north–south axis of the country [33]. Correspondingly, different epizootiological profiles were displayed throughout the country in another survey, where the prevalence rate ranged from 6.5% to 50.2%, depending on the prefecture [31]. This distribution pattern has been noted in other endemic areas [26] and has been attributed to several factors, such as variant climatic and socioeconomic conditions of regions, as well as the presence of *Phlebotomus* and canine populations [40].

The above data have been confirmed by epizootiological studies that employed serology as a diagnostic approach. Serological assays are labour-saving, cost-effective, and can be performed rapidly on-site [18]. In addition, they are specific and are considered efficient for assessing the distribution of seropositive animals [41,42]. Moreover, they can detect the majority of symptomatic and a proportion of asymptomatic dogs [41,43,44]. However, serological investigation fails to identify infected dogs that will never seroconvert [16,45] or dogs during the prepatent period before seroconversion [45,46] as well as those that are seropositive and seroreverted while at the same time remaining parasite positive [16,47]. Therefore, serological screening underestimates the true number of infected dogs in endemic areas since a much higher proportion of dogs is actually infected than that recorded with the diagnosis mediated by serology [43]. The actual *L. infantum* infection rate can be precisely estimated using immunoblotting and molecular techniques [36,48] or cell-mediated assays [35]. Towards this end, a study in Greece using polymerase chain reaction (PCR) and indirect immunofluorescence assay (IFAT) examined 73 clinically normal dogs with an outdoor lifestyle and recorded a prevalence of 63% and 12.2%, respectively, concluding that most of the dogs residing in Greece are infected [19]. To be accurate, the estimates documented in sero-epizootiological surveys are expected to be somewhere between the observed prevalence of disease and the actual infection rate [1,36,48].

As regards the possible factors that may amplify the risk of infection in Greece, an updated multicenter study highlighted that the outdoor lifestyle of a dog, increased environmental humidity and annual rainfall, as well as decreased ambient mean temperature and mean wind speed, were positively correlated with the probability of acquiring the infection. On the contrary, gender, age, dog breed, and altitude of a region were not associated with canine seropositivity for *L. infantum* [33]. In a previous study conducted in Central Greece, the maximum temperature of the warmest month was identified as a significant contributing variable in canine prediction models, probably due to its strong effect on vector distribution and activity [49]. It seems that there is no direct correlation between dog and human seropositivity, as it has been indicated in a seroprevalence study of dogs and humans that co-exist in the same endemic area of Greece, i.e., the seropositivity levels of dogs and humans were 48% and 1.5%, respectively [50].

## 4. Prevalence of *L. infantum* in the Feline Population in Greece

Cats may be involved in the transmission cycle of *L. infantum*, more likely as secondary reservoirs [51,52,53]. Following experimental infection with the parasite, it has been evidenced that they are less susceptible than dogs [51,52], which display a higher parasitic burden under the same natural setting [54]. Therefore, the disease manifests scarcely in this animal species, although some infected cats may develop clinical signs and/or laboratory abnormalities of leishmaniosis [55,56]. Nonetheless, in endemic areas, cats are expected to be challenged with the parasite, become infected, and serve as a persisting source of infection for vectors [53]. The ability of phlebotomine sandflies to feed on cats and acquire *L. infantum* has been indirectly documented in experimental studies [51,57,58]. The above, coupled with the fact that there is an abundance of outdoor living cats in Greece, suggest that their role in the maintenance and transmission of this protozoon in the country could be more critical than speculated.

Albeit the prevalence of feline infection is generally lower than that in dogs from the same localities, the percentage of infected cats is not negligible [56,59,60]. Several epizootiological studies have demonstrated that the parasite circulates among feline populations in Mediterranean countries [59,60,61,62,63,64,65]. Studies in Greece have evaluated the exposure to *L. infantum* and have recorded a seroprevalence rate of 2.0% in Mykonos island [66], 3.8% in Thessaloniki [59], 8.3% in Athens and 14.7% in Crete island [63]. In the same context, a comprehensive multicenter survey about feline leishmaniosis in the Mediterranean basin recorded 8.1% seropositivity in Greek cats [65], and a meta-analysis review of the literature has demonstrated a seroprevalence rate of 11.0% in cats in Greece [67]. Using molecular techniques, *Leishmania* spp. DNA was identified in 13.3% of cats’ blood samples from Central Greece [68]. Another study conducted in Central and Northern Greece that tested multiple tissue samples recorded 41% and 40% PCR positivity in clinically normal cats and cats with various clinical signs, respectively [56]. As in the case of dogs, it has been documented that feline leishmaniosis may be imported in non-endemic areas from cats that originate from endemic areas or that have travelled with their owners in such spots [69,70]. The aforementioned data suggest that cats may serve as a reservoir for *L. infantum* and further enhance infection chances for dogs and other hosts in Greece.

## 5. Prevalence of *L. infantum* in Other Host Populations in Greece

Other mammals, such as rodents, lagomorphs, minks and equines, can act as alternative reservoir hosts for the parasite and can contribute to maintaining the infection in nature. In a survey carried out in Athens and Piraeus in wild *Rattus norvegicus,* one positive spleen sample out of the sixteen assayed by PCR for *L. infantum* was detected [71], whereas in another larger scale study, *Leishmania* spp. DNA was present in 19.6% of the examined rodents (*n* = 97) [72]. The same study depicted specific anti-*Leishmania* IgG in 54.5% of the sampled animals [72]. Regarding lagomorphs, the *L. infantum* DNA prevalence in European brown hares (*Lepus europaeus*) originating from Northern Greece was reported to be 23.49% during 2007–2011, and the subsequent phylogenetic analysis revealed that all sequences belonged to the *L. donovani* complex [73]. Likewise, a later study in hares sampled in Northern Greece showed a *L. infantum* DNA prevalence of 20%, while a lower percentage of PCR-positive animals (7.4%) was recorded in Central Greece [74]. A total seroprevalence of 12.4% was reported in the same hare population [74]. Both studies identified precipitation seasonality as a critical influential environmental parameter associated with *L. infantum* infection in hares, probably due to its effects on the sandfly vector activity, survival, and oviposition sites [73,74]. Another serological survey of samples originating from areas of Northern Greece revealed infection in 7.6% of rabbits (*Oryctolagus cuniculus*), 6.7% of hares, and 20% of minks (*Mustela vison*) [75]. Minks displayed the highest seropositivity among the species examined. Interestingly, *L. infantum* infection and the subsequent immunosuppression have been linked to a severe outbreak of hemorrhagic pneumonia due to *Pseudomonas aeruginosa* in a Greek mink farm [76]. Concerning horses, antibodies to *Leishmania* spp. were present (0.3%) only in animals originating from an equestrian centre located in the Attica region [77]. All these natural hosts are of particular interest, and additional studies are in demand to clarify whether they can be reservoirs and their role in the epizootiological cycle of the parasite.

## 6. Prevalence of *Leishmania* Infection in Sandflies and the Sandfly Fauna in Greece

Several entomological studies in Greece have provided valuable data about sandfly distribution, abundance, and taxonomy in the mainland and the Greek islands. Up to now, two sandfly species belonging to the genus *Sergentomyia*—*Sergentomyia dentata* and *S. minuta*—and eleven species belonging to the medically important genus *Phlebotomus* have been recorded—*Phlebotomus perfiliewi*, *P. neglectus*, *P. tobbi*, *P. balcanicus*, *P. simici*, *P. papatasi*, *P. sergenti*, *P. similis*, *P. alexandri*, *P. mascittii*, and the newly described *P.* (*Adlerius*) *creticus* n. sp., which has been identified in various localities on the island of Crete [78,79,80,81,82]. Among the different species mentioned above, the proven or suspected vectors of *Leishmania* spp. are *P. perfiliewi*, *P. tobbi*, and *P. neglectus* for *L. infantum*, *P. similis* for *L. tropica* and *P. papatasi* for *L. major* [81,83,84,85,86]. Monitoring sandfly fauna and its infection prevalence for the different *Leishmania* spp. is of great significance, as sandflies are the only proven vectors of this protozoan parasite [87].

In that context, a study conducted in urban, periurban, and rural areas of Attiki, Central Greece, during four consecutive years revealed a 0.41% prevalence of *L. infantum* DNA in the examined sandflies [83]. Higher sandfly abundance was recorded in periurban areas, from June to September, in areas with moderate to high temperatures and the highest altitude of 429 m [83]. Among the seven different sandfly species identified (*P. tobbi*, *S. minuta*, *P. neglectus*, *P. simici*, *P. papatasi*, *P. similis*, and *P. alexandri*), the most prevalent was *P. tobbi* (41.52%) followed by *S. minuta* (27.44%), *P. neglectus* (14.83%) and *P. simici* (11.08%) [83]. However, a previous study in the Attica region reported a higher infection prevalence of 5.4% [88]. It has been suggested that this difference may be attributed to the different study designs and the molecular tool used in the latter study (semi-nested PCR assay) [83]. An earlier study on the island of Corfu demonstrated a low *L. infantum* infection prevalence of 0.12% in *P. neglectus* [89]. In that study, culture was used as a means of detection and typing of *Leishmania* spp. [89].

In a 2019 study, the researchers detected *L. donovani* and *L. tropica* DNA in naturally infected sandflies collected during 2017 in two refugee camps in the Thessaloniki region in Northern Greece [90]. Phylogenetic analysis revealed that the detected *L. donovani* and *L. tropica* sequences clustered with haplotypes mainly from Syria, Iraq, India, Sudan, and Ethiopia, which have been associated with human visceral leishmaniosis [90]. The prevalence of infection was unusually high in all three sandfly species identified (*P. perfiliewi*, *P. tobbi*, *P. simici*), ranging from 43% to 52% in the two refugee camps [90]. This study highlighted the high levels of focal parasite transmission in the Thessaloniki refugee camps and the increased risk of infection within refugee populations and the surrounding communities [90]. *L. donovani*—causing both anthroponotic visceral and cutaneous leishmaniosis—was recently introduced in the Mediterranean basin [91], and to the best of our knowledge, there are no records of the circulation of this species in Greece. The continuous surveillance of the sandfly fauna is currently imperative for establishing and implementing strong and effective strategies against canine and human leishmaniosis.

## 7. Conclusions

*Leishmania infantum* circulates among canine populations in Greece and remains challenging for practitioners. Greek cats may be at risk of infection and clinical disease manifestation, while their epizootiological role in maintaining and transmitting the parasite requires further corroboration. Updating the epizootiological profile of the infection in Greece is vital given the country’s touristic appeal; therefore, further studies are warranted. This update would assist in the early diagnosis and implementation of targeted effective control measures for the subsequent protection of animal and human health.

## Data Availability

Not applicable.

## Abbreviations

CanL: canine leishmaniosis; *L. infantum*: *Leishmania infantum*; *L. donovani*: *Leishmania donovani*; PCR: polymerase chain reaction; IFAT: indirect immunofluorescence assay.
